# Editorial: Future Perspectives of Sentinel Node Mapping in Gynecological Oncology

**DOI:** 10.3389/fonc.2022.809765

**Published:** 2022-02-25

**Authors:** Angela Santoro, Frediano Inzani, Giuseppe Angelico, Fabio Martinelli, Andrea Papadia, Gian Franco Zannoni

**Affiliations:** ^1^ Unità di Ginecopatologia e Patologia Mammaria, Dipartimento Scienze della Salute della Donna, del Bambino e di Sanità Pubblica, Fondazione Policlinico Universitario A. Gemelli IRCCS, Roma, Italy; ^2^ Gynecologic Oncology Unit, Fondazione Istituto Nazionale Tumori Istituto di Ricerca e Cura a Carattere Scientifico (IRCCS), Milan, Italy; ^3^ Department of Obstetrics and Gynecology, University Hospital of Bern and University of Bern, Bern, Switzerland; ^4^ Department of Gynecology and Obstetrics, Ente Ospedaliero Cantonale of Lugano, University of the Italian Switzerland (USI), Lugano, Switzerland; ^5^ Istituto di Anatomia Patologica, Università Cattolica del Sacro Cuore, Roma, Italy

**Keywords:** Research Topic editorial, sentinel node mapping, gynecological oncology, prognosis, women health

Lymph node (LN) metastasis in gynecological malignancies represents the most important negative prognostic predictor (1). Considering the high treatment-related morbidity of regional lymphadenectomy, sentinel LN (SLN) mapping has been increasingly used in the last years for staging purposes in gynecological tumors (1, 2). Clinical and pathological aspects specific to each gynecological site are discussed below.

In breast cancer the presence of metastatic SLNs still necessitates the recommended procedure for axillary staging of early neoplasms (3). Currently, circulating microRNAs (miRNAs) are recognized as promising non-invasive biomarkers and innovative prognostic factors, being correlated to LN status, occurrence of distant metastases, and recurrence. In their work, Escuin et al. showed the different expression profile of several circulating miRNAs in relation to the SLN status, suggesting the potential role of peripheral blood circulating nucleic acids as surrogate markers of LN metastases in early breast cancer patients.

SLN mapping in early-stage vulvar cancer represents the gold standard for patients with unifocal vulvar tumor, >1 mm in thickness and negative groin lymph nodes by clinical and imaging examination (Zhou et al., Siegenthaler et al.). Since the SLN utility and applications in vulvar cancer are still debated, Zhou et al. compared the safety of SLN biopsy (SLNB) with regional LN dissection (RLND) in patients with vulvar squamous cell cancer (Zhou et al.). Their findings indicate that SLNB is related to prolonged survival outcomes in patients with no metastatic or advanced-stage disease compared to RLND and no LN removed, irrespective of tumor size, surgery type, or invasion depth (Zhou et al.). A recent study has also established the clinical utility of ultrasound-guided fine-needle aspiration cytology (FNAC), stating that a positive result is sufficient to avoid an unnecessary SLN sampling, enabling the surgeon to perform a bilateral inguinofemoral lymphadenectomy (4).

The standard SLN detection technique involves a peritumoral injection of technetium-99m (99mTc) nanocolloid combined with an intraoperative injection of a blue dye. A novel, potentially interesting, SLN mapping technique has been proposed by Siegenthaler et al., demonstrating an improvement of the SLN detection rate by using a combination of ^99m^Tc-nanocolloid with indocyanine green.

Regarding SLN mapping in cervical cancer, the New ESGO/ESTRO/ESP guidelines incorporated SLN biopsy as an acceptable method of LN staging in early-stage cervical cancers, particularly in cases of small volume tumors (5). In the light of preliminary results of ongoing trials, and considering the long-term morbidity related to full pelvic LN dissection, a minimally invasive approach represents the standard of care in early-stage cervical cancer patients (5, 6). In this regard, Favre et al. in their randomized study, comparing early-stage patients undergoing SLN biopsy alone versus pelvic LN dissection, demonstrated no significant differences between the two groups in terms of overall survival and disease recurrence.

The gold-standard technique to process SLN in cervical cancer is the ultrastaging protocol (1, 7).

This technique, requiring LNs serial sectioning and immunohistochemistry, is utilized in pathology laboratories to confirm the negative status of a LN and also detect small-volume metastases, ranging between 0.2 and 2 mm in size. Recently, OSNA protocol, based on a quantitative measurement of target mRNA in a metastatic LN, has been proposed as an efficient alternative method for the intra-operative assessment of SLN in cervical cancer patients (8, 9). To date, the biological significance of small volume metastases in early cervical cancer is still highly debated. In the most detailed studies available, the presence of SLN micrometastases is related with a worse prognosis, representing an indication for adjuvant radiotherapy (1, 7). However, recently, SENTICOL1 trial results showed that SLNs micrometastases did not impact on progression-free survival in cervical cancer patients (10). Similarly, the biological significance and clinical management of SLN isolated tumor cells (ITCs) is still highly debated (1, 7).

As illustrated in the review article by Zhai et al., in endometrial cancer SLN mapping has emerged as a reliable alternative to pelvic LN dissection. Several studies have demonstrated a high sensitivity and negative predictive value to detect nodal metastases leading to similar oncological outcomes between patients undergoing SLN and pelvic lymphadenectomy (1, 2, Zhai et al.). Therefore, in 2020 National Comprehensive Cancer Network (NCCN) guidelines, SLN mapping has been recommended for staging purposes in EC patients (1). In this regard, according to the recent meta-analysis conducted by Gu et al., SLN mapping seems the more appropriate approach for both low- and high-risk EC patients given its lower surgical risk and patient morbidity in comparison to pelvic LN dissection. However, the article by Pineda et al., highlighted that high-risk EC patients could still benefit from pelvic LN dissection since sensitivity and negative predictive value observed in their cohort were 85.7 and 96.6% respectively (Gu et al.). On the other hand, SLN biopsy demonstrated high sensitivity and negative predictive values in intermediate-risk EC patients (Gu et al.).

Therefore, according to available literature data, full lymphadenectomy could be avoided by performing SLNB in patients of low and intermediate risk while additional data on larger cohorts need to be collected in order to demonstrate the staging and prognostic benefits of SLN biopsy in high-risk EC.

LN status is also a relevant prognostic factor in early ovarian carcinoma; however, para-aortic and pelvic lymphadenectomies carry a significant risk of intra- and post-operative morbidity (11, 12). SLN mapping could represent a safer staging alternative to lymphadenectomy, however studies on this topic are still limited to small cohorts. Preliminary results of a currently opened prospective multicenter study (SELLY: Sentinel-node biopsy in early-stage ovarian cancer: preliminary results) suggest that SLN can be difficult to identify even for experienced surgeons (12). Moreover, the authors pointed out that larger cohort studies are needed in order to determine the real sensitivity and negative predictive value of this technique (12).

In conclusion, SLN biopsy is an intraoperative procedure with potential for adequate staging with less treatment-related morbidity. It should be performed by a skilled multidisciplinary team, in oncology centers, preferably within the protection of clinical trials. Different methods of histopathological and molecular SLN assessment according to the different gynecological cancers have been proposed. This Research Topic, collecting several papers on this topic, discusses the need for standardization of pathological protocols, the molecular aspects of SLN evaluation in gynecological cancer, and the clinical benefits of this treatment option in routine practice.

## Author Contributions

All authors contributed to the Research Topic editorial and performed the literature search. AS and GA drafted the manuscript. GZ critically revised the work. All authors read and approved the final manuscript.

## Conflict of Interest

The authors declare that the research was conducted in the absence of any commercial or financial relationships that could be construed as a potential conflict of interest.

## Publisher’s Note

All claims expressed in this article are solely those of the authors and do not necessarily represent those of their affiliated organizations, or those of the publisher, the editors and the reviewers. Any product that may be evaluated in this article, or claim that may be made by its manufacturer, is not guaranteed or endorsed by the publisher.
